# Differential changes in amygdala and frontal cortex *Pde10a* expression during acute and protracted withdrawal

**DOI:** 10.3389/fnint.2014.00030

**Published:** 2014-04-08

**Authors:** Marian L. Logrip, Eric P. Zorrilla

**Affiliations:** Committee on the Neurobiology of Addictive Disorders, The Scripps Research InstituteLa Jolla, CA, USA

**Keywords:** alcohol or ethanol dependence, basolateral or central or medial nucleus of the amygdala, alcoholism or alcohol use disorder, acute or protracted withdrawal or abstinence, phosphodiesterase 10A, medial prefrontal or infralimbic or anterior cingulate cortex, dorsal striatum

## Abstract

Alcohol use disorders are persistent problems with high recidivism rates despite repeated efforts to quit drinking. Neuroadaptations that result from alcohol exposure and that persist during periods of abstinence represent putative molecular determinants of the propensity to relapse. Previously we demonstrated a positive association between phosphodiesterase 10A (PDE10A) gene expression and elevations in relapse-like alcohol self-administration in rats with a history of stress exposure. Because alcohol withdrawal is characterized by heightened anxiety-like behavior, activation of stress-responsive brain regions and an elevated propensity to self-administer alcohol, we hypothesized that *Pde10a* expression also would be upregulated in reward- and stress-responsive brain regions during periods of acute (8–10 h) and protracted (6 weeks) alcohol withdrawal. During acute withdrawal, elevated *Pde10a* mRNA expression was found in the medial and basolateral amygdala (BLA), as well as the infralimbic and anterior cingulate subdivisions of the medial prefrontal cortex, relative to alcohol-naïve controls. The BLA was the only region with elevated *Pde10a* mRNA expression during both acute and protracted withdrawal. In contrast to the elevations, *Pde10a* mRNA levels tended to be reduced during protracted withdrawal in the dorsal striatum, prelimbic prefrontal cortex, and medial amygdala. Together these results implicate heightened PDE10A expression in the BLA as a lasting neuroadaptation associated with alcohol dependence.

## INTRODUCTION

Phosphodiesterase 10A (PDE10A) is a dual-specificity phosphodiesterase, a family of enzymes that regulates cyclic nucleotide activity to modulate intracellular signaling pathways (Francis et al., 2011). PDE10A can hydrolyze both cyclic adenosine monophosphate (cAMP) and cyclic guanosine monophosphate (cGMP; Fujishige et al., 1999a; Loughney et al., 1999; Soderling et al., 1999) and is prominently expressed in the brain, particularly in the striatum (Fujishige et al., 1999b; Seeger et al., 2003). Like other PDEs, PDE10A may play an important role in neuronal plasticity by modulating the levels of active cAMP and cGMP available to participate in intracellular signaling cascades (Kroker et al., 2012; Wiescholleck and Manahan-Vaughan, 2012; Zhong et al., 2012; Uthayathas et al., 2013). Acute inhibition of PDE10A increased striatal neuronal activity following cortical stimulation (Threlfell et al., 2009), and chronic PDE10A inhibition or genetic deletion altered the expression of several genes encoding proteins involved in neurotransmission (Kleiman et al., 2011). Additionally, hippocampal long-term potentiation increased the expression of several splice variants of *Pde10a* (O’Connor et al., 2004), suggesting that altered PDE10A levels may help subserve long-term memory formation. Importantly, PDE10A has been implicated in both appetitive and aversive conditioning (Piccart et al., 2011, 2013), as well as in regulating striatal dopaminergic responses to amphetamine (Sotty et al., 2009). Taken together, these data suggest key roles for PDE10A in reward-related learning and neural responses to reinforcers, including drugs of abuse.

Substance use disorders have been conceptualized as diseases of aberrant plasticity (Kauer and Malenka, 2007) in which repeated drug or alcohol exposure alters the hedonic set-point. In the resulting allostatic state, drugs, or alcohol are consumed to alleviate or prevent aversive withdrawal symptoms rather than for positive reinforcing effects (Koob and Le Moal, 2001). The negative emotional state that arises during acute withdrawal from alcohol exposure includes elevations in anxiety-like behavior (Baldwin et al., 1991; Knapp et al., 1998; Pandey et al., 1999; Valdez et al., 2002), which subside over the first few days after removal of alcohol access. However, a resurgence of heightened anxiety-like behavior (Zhao et al., 2007) and increased sensitivity to stressors (Valdez et al., 2002; Sommer et al., 2008) have been reported in rats during protracted periods of alcohol withdrawal, weeks or months after the final exposure to alcohol. Such lasting negative emotional symptoms are hypothesized to motivate relapse (Koob and Le Moal, 2001). Accordingly, molecular neuroadaptations that are present during both acute and protracted withdrawal may have key roles in the long-term propensity for abstinent individuals to relapse (Dawson et al., 2007) and represent targets for pharmacotherapeutic development. Because the negative emotional state of alcohol withdrawal is characterized by reduced reward function (Schulteis et al., 1995), PDE10A is a candidate for withdrawal-induced neuroadaptation based on its prominent localization in and ability to regulate neuronal activity in reward-responsive brain regions (Threlfell et al., 2009; Mango et al., 2014). A role for PDE10A in regulating behavioral responses to stress also is supported by findings that genetic (Siuciak et al., 2006b) or pharmacological (Siuciak et al., 2006a; Schmidt et al., 2008; Grauer et al., 2009) reduction of PDE10A activity in rats and mice reduces conditioned avoidance of a shock-paired chamber.

Recently we observed a relationship between *Pde10a* mRNA levels and relapse-like alcohol self-administration in rats with a history of stress exposure (Logrip and Zorrilla, 2012). Rats with a history of stress demonstrated elevated *Pde10a* expression in the basolateral amygdala (BLA) and heightened relapse-like alcohol self-administration. Furthermore, in rats with a stress history, *Pde10a* mRNA levels in the infralimbic and prelimbic prefrontal cortices (plPFCs) correlated with greater alcohol intake, and the prelimbic cortex showed increased *Pde10a* mRNA levels vs. unstressed controls in the group with elevated relapse-like self-administration. The data implicate PDE10A as a locus for neuroadaptation that regulates behavioral responses to stress, including elevated alcohol intake. Therefore, in the present study we hypothesized that *Pde10a* expression also would be elevated during acute and/or protracted alcohol withdrawal, periods of elevated anxiety-like behavior (Valdez et al., 2002; Zhao et al., 2007) and heightened alcohol intake potential (Valdez et al., 2002). In particular, we hypothesized those changes in *Pde10a* expression would most likely occur in brain nuclei involved in both reward and stress responses.

## MATERIALS AND METHODS

### SUBJECTS

Adult male Wistar rats, 175–200 g upon arrival, were obtained from Charles River Laboratories (Bar Harbor, ME, USA) and housed three per cage upon arrival. Rats were housed in a temperature- and humidity-controlled vivarium under a reversed light cycle (lights on 8 pm–8 am), with food (Harlan Teklad LM-485, Indianapolis, IN, USA) and water available *ad libitum*. Procedures were approved by the Institutional Animal Care and Use Committee of The Scripps Research Institute and conformed to guidelines set forth in the National Institutes of Health Guide for the Care and Use of Animals.

### ALCOHOL VAPOR EXPOSURE

Rats designated for intermittent alcohol vapor exposure were transferred to housing in alcohol vapor chambers, where they were provided with alcohol vapor in the air supply (Gilpin et al., 2008) for 14 h daily over 5 weeks. Daily alcohol exposures were followed by a 10-h withdrawal period, yielding daily cycles of intoxication/withdrawal. Vapor exposure levels were regulated on a weekly basis to maintain blood alcohol levels (BALs) between 175 and 225 mg%. Serum BALs were determined using the Analox AM1 Alcohol Analyzer (Analox Instruments USA Inc., Lunenberg, MA, USA), with tail blood samples collected weekly during the final 2 h of the 14-h vapor exposure period.

### TISSUE PUNCH COLLECTION

Rats were euthanized during acute (8–10 h after vapor shutoff) or protracted (6 weeks after cessation of intermittent vapor exposure) withdrawal via rapid decapitation under isoflurane anesthesia. Using a wire matrix, 2 mm coronal brain slices were obtained and immediately immersed in RNAlater (Qiagen Inc., Valencia, CA, USA). Regions of interest were punched on a chilled stage using 14-gage (prefrontal cortex and striatal sections) and 18-gage (amygdala subdivisions) blunt needles, then stored at -80°C for subsequent processing.

### RNA EXTRACTION, REVERSE TRANSCRIPTION, AND QUANTITATIVE PCR ANALYSIS

Ribonucleic acid samples were processed and quantified as in (Logrip and Zorrilla, 2012). Briefly, RNA was extracted with QIAzol (Qiagen, Inc., Valencia, CA, USA), samples were treated with DNase I (EMD Millipore, San Diego, CA, USA) to remove genomic DNA contamination, and concentrations were determined using the Quant-iT RiboGreen RNA Assay Kit (Invitrogen, Carlsbad, CA, USA). cDNA was reverse transcribed using the Superscript III First Strand Synthesis System (Invitrogen, Carlsbad, CA, USA) with Oligo (dT)_20_ primers. Gene expression levels were assessed by quantitative polymerase chain reaction (qPCR) using Light Cycler 480 SYBR Green I (Roche Applied Science, Indianapolis, IN, USA) and 0.5 μM primers per reaction (ValueGene Inc., San Diego, CA, USA), with sequences and primer conditions as previously described (Sabino et al., 2009; Logrip and Zorrilla, 2012). Reactions were run on a Mastercycler ep realplex^4^ thermal cycler (Eppendorf North America, Hauppauge, NY, USA). Threshold cDNA copy number was interpolated per standard curves of purified PCR product, and results were analyzed via second derivative methods. C*yclophilin A* (*Cyp*) was used as the internal standard.

### WESTERN BLOTTING

Tissue samples were homogenized in TEVP buffer [10 mM Tris base, 5 mM sodium fluoride, 1 mM sodium orthovanadate, 1 mM ethylenediaminetetraacetic acid, 1 mM ethylene glycol-bis(2-aminoethylether)-N,N,N′,N′-tetraacetic acid, pH 7.4] containing 320 mM sucrose and cOmplete Protease Inhibitor Cocktail (Roche Diagnostics Corporation, Indianapoils, IN, USA) using a rotor-stator homogenizer (Tissue Tearor, Cole-Parmer Instrument Co., Vernon Hills, IL, USA). A centrifugal fractionation procedure was utilized to enrich postsynaptic density-containing membranes according to the method of (Hallett et al., 2008). Protein samples obtained pre-fractionation (total lysate, 3 μg) and the fraction obtained after hypoosmotic lysis in TEVP buffer with 36 mM sucrose and subsequent 21,000 × *g* centrifugation (postsynaptic density-enriched membrane fraction, BLA: ~4 μg, striatum: 5 μg) were subjected to polyacrylamide gel electrophoresis (4–15% Tris-HCl Ready Gel, Bio-rad Laboratories, Inc., Hercules, CA, USA, or 4–12% Invitrogen BOLT Bis-Tris Plus gels, Life Technologies, Grand Island, NY, USA). Proteins were transferred to PVDF membranes (Immobilon-P, EMD Millipore, Billerica, MA, USA) using standard Western blotting techniques and blocked in 5% Blotting-Grade Blocker (Bio-rad) in Tris-buffered saline containing 0.1% Tween-20 (Sigma-Aldrich, Inc., St. Louis, MO, USA) prior to incubation in rabbit anti-PDE10A or mouse anti-β-actin primary antibodies [Sigma-Aldrich; SAB2700582, 1:250 (BLA) or 1:500 (striatum); A2228, 1:25,000]. Following incubation in HRP-conjugated secondary antibodies [goat anti-mouse (Bio-rad, 1706516, 1:25,000) and donkey anti-rabbit (EMD Millipore, AP182PMI, 1:15,000)], proteins were visualized via enhanced chemiluminescence (SuperSignal West Pico, Thermo Scientific Pierce, Pittsburgh, PA, USA) and exposure to Hy-Blot CL film (Denville Scientific, South Plainfield, NJ, USA). Digital images were acquired using light transmission scanning on the Scanjet G4050 (Hewlett-Packard Company, Palo Alto, CA, USA) and band intensity quantified using ImageJ software (National Institutes of Health, Bethesda, MD, USA; Schneider et al., 2012).

### STATISTICAL ANALYSIS

Quantitative polymerase chain reaction data were normalized using *z*-scores and analyzed by ANCOVA with *Cyp* expression levels controlled as a covariate. Because skewing by outliers can generate false positive results with ANCOVA analysis, two criteria were applied to identify outliers causing significant alteration in the results. First, any samples with *Cyp* values more than 3 standard deviations from the mean of all samples were excluded (Pukelsheim, 1994), and similarity of *Cyp* values between treatment groups was confirmed by Student’s *t*-test (*p* > 0.1). Second, any data causing excessive skew of the regression, defined as observations with studentized residuals greater than 3 for which also either the Cook’s *D* exceeded the value required to yield *p* < 0.50 (Cook, 1977) or the leverage was more than twice the treatment mean, were excluded from the analysis (Belsley et al., 1980). The collective outlier criteria resulted in exclusion of 5.9% of all samples. Statistical analyses were performed using Systat 12.0 (Chicago, IL, USA). Bonferroni-corrected *post hoc* pairwise comparisons of ANCOVA-generated least squares means were performed by GraphPad QuickCalcs (http://graphpad.com/quickcalcs/posttest1.cfm) as needed.

## RESULTS

### MEDIAL PREFRONTAL CORTEX *Pde10a* mRNA IS DIFFERENTIALLY ALTERED DURING ACUTE vs. PROTRACTED WITHDRAWAL

**FIGURE 1:**
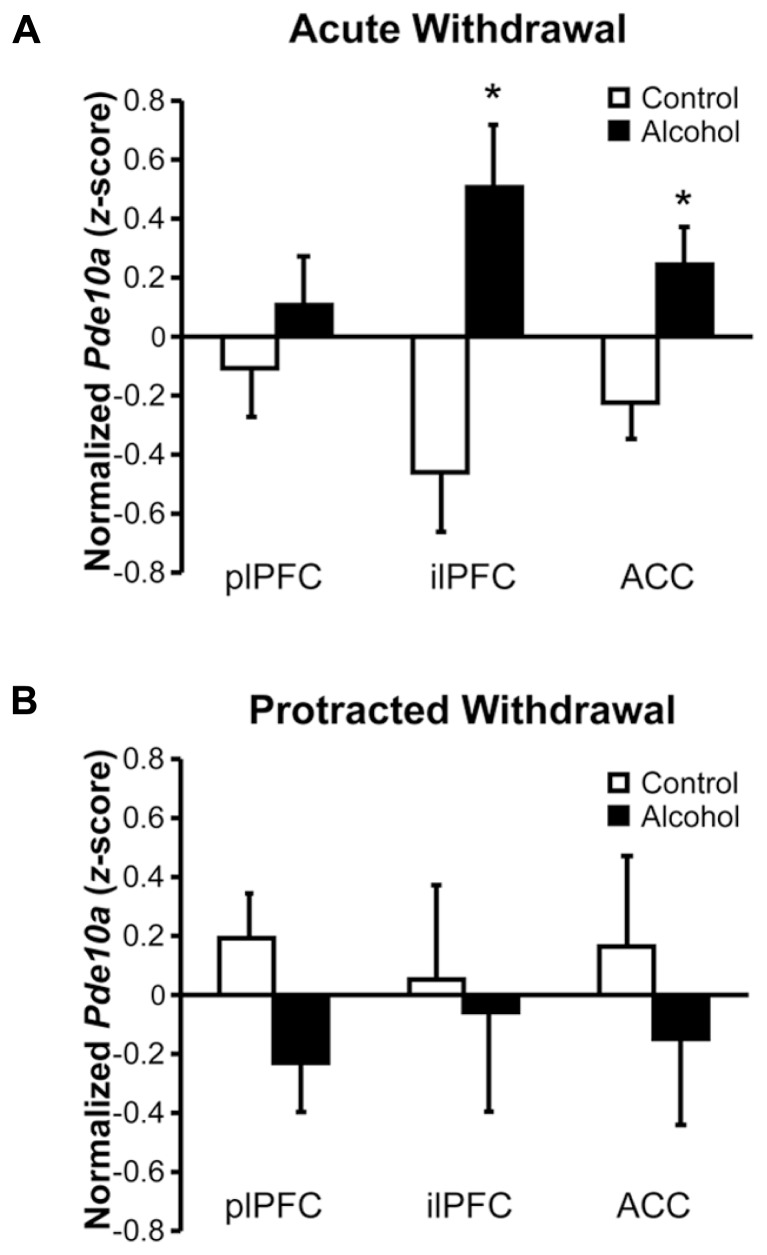
**Acute and protracted alcohol withdrawal differentially impacted *Pde10a* mRNA expression in the medial prefrontal cortex**. **(A)** Acute (8–10 h) withdrawal from intermittent alcohol vapor (black bars) exposure significantly increased *Pde10a* mRNA levels in the infralimbic (ilPFC) and anterior cingulate (ACC), but not prelimbic (plPFC), subdivisions of the medial prefrontal cortex, relative to alcohol-naïve control rats (white bars). **(B)** During protracted withdrawal, 6 weeks after the last alcohol vapor exposure, *Pde10a* mRNA expression tended to be decreased in the plPFC but was not significantly altered in the ilPFC or ACC. Data are presented as least squares means ± standard error of *z*-score transformed *Pde10a* covaried for *Cyp* expression levels. *p < 0.05 vs. Control; *n* = 9–12 per group.

Rats withdrawn from chronic intermittent alcohol vapor exposure showed subregion-specific alterations in *Pde10a* mRNA expression in the medial prefrontal cortex (mPFC; **Figure 1**). As shown in **Figure 1A**, *Pde10a* mRNA levels were significantly elevated in the infralimbic (ilPFC; *F*_1,18_ = 10.84, *p* < 0.005) and anterior cingulate (ACC; *F*_1,20_ = 6.89, *p* < 0.05) subdivisions of the mPFC during acute alcohol withdrawal. However, these alterations in *Pde10a* mRNA levels did not persist into protracted withdrawal (**Figure 1B**), as neither ilPFC (*F*_1,16_ = 0.05, *p* = 0.82) nor ACC (*F*_1,18_ = 0.52, *p* = 0.48) *Pde10a* expression levels differed from alcohol-naïve controls at the later withdrawal time point. Conversely, no significant alteration in *Pde10a* levels was seen in the plPFC during acute withdrawal (**Figure 1A**; *F*_1,21_ = 0.84, *p* = 0.37), whereas a trend toward reduced expression in the plPFC was found during protracted withdrawal (**Figure 1B**; *F*_1,19_ = 3.41, *p* = 0.08).

### UPREGULATED AMYGDALA *Pde10a* mRNA PERSISTS IN THE BASOLATERAL NUCLEUS OF THE AMYGDALA

**FIGURE 2:**
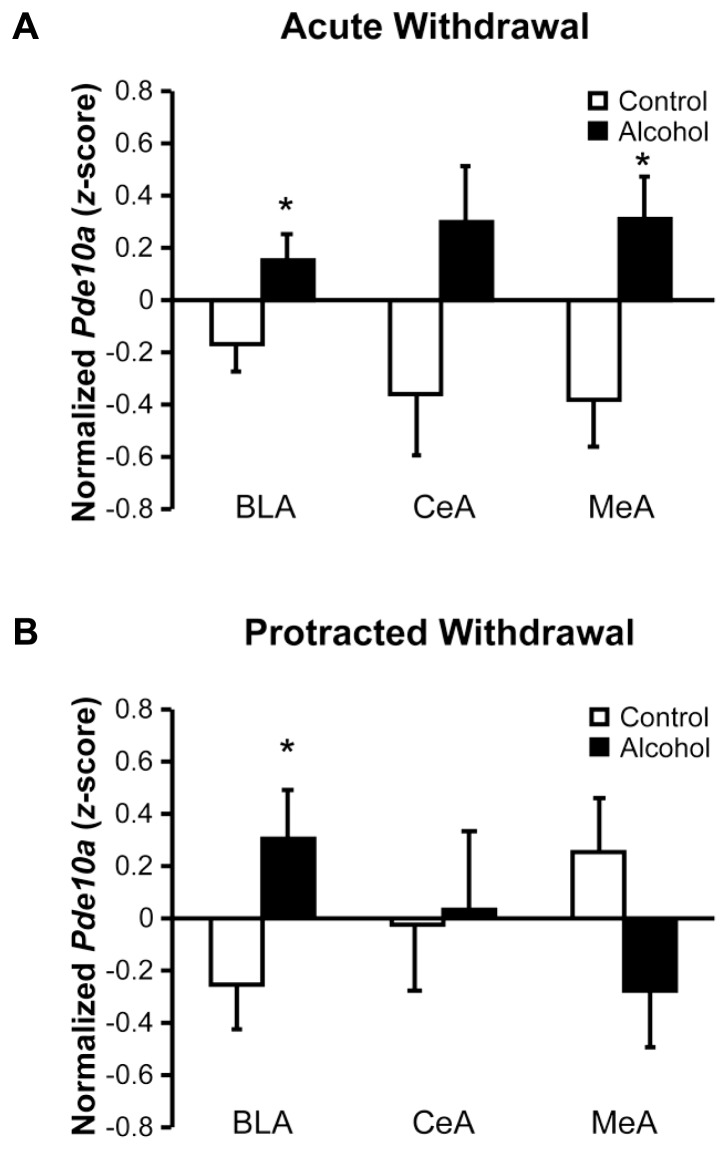
**Acute and protracted alcohol withdrawal generated lasting increases in *Pde10a* mRNA expression in the basolateral amygdala, but opposite profiles in the medial amygdala**. **(A)** Acute (8–10 h) withdrawal from intermittent alcohol vapor (black bars) exposure significantly increased *Pde10a* mRNA levels in the basolateral (BLA) and medial (MeA) nuclei of the amygdala, without significant alteration in the central nucleus (CeA), relative to alcohol-naïve control rats (white bars). **(B)** *Pde10a* mRNA levels remained elevated in the BLA 6 weeks later, during protracted withdrawal, while expression levels tended to be decreased in the MeA. No significant difference between control and alcohol-withdrawn rats was observed in the CeA. Data are presented as least squares means ± standard error of *z*-score transformed *Pde10a*, covaried for *Cyp* expression levels. *p < 0.05 vs. Control; *n* = 8–12 per group.

Increased *Pde10a* expression was observed throughout the amygdala during acute withdrawal (**Figure 2A**), with significant elevations seen in the basolateral (BLA; *F*_1,20_ = 4.85, *p* < 0.05) and medial (MeA; *F*_1,17_ = 7.90, *p* < 0.05) nuclei, and a trend for an increase seen in the central nucleus (CeA; *F*_1,19_ = 4.27, *p* = 0.053). During protracted withdrawal, *Pde10a* mRNA was still elevated in the BLA (**Figure 2B**; *F*_1__,__19_ = 4.74, *p* < 0.05) but not the CeA (*F*_1,16 _= 0.02, *p* = 0.89), whereas a slight trend toward decreased *Pde10a* expression was seen in the MeA (*F*_1,20 _= 2.99, *p* = 0.099).

### ALCOHOL WITHDRAWAL DOES NOT ACUTELY MODIFY STRIATAL OR SEPTAL *Pde10a* EXPRESSION

**FIGURE 3:**
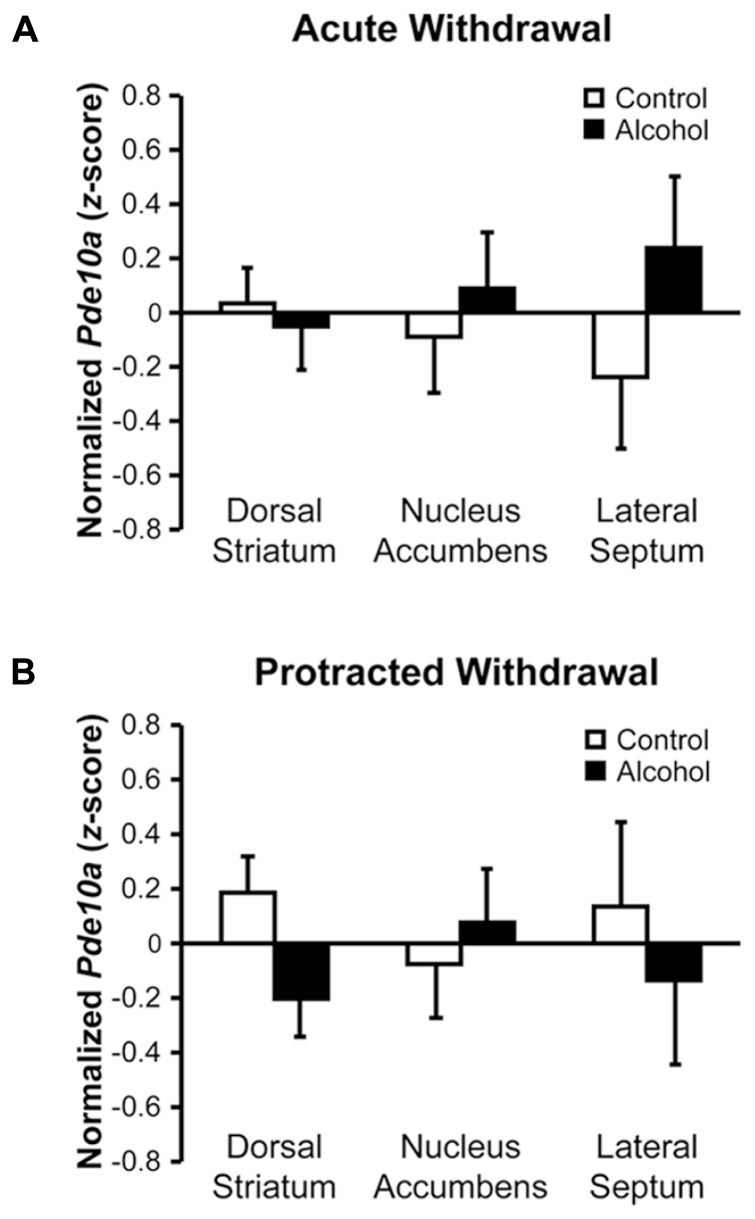
**Acute and protracted withdrawal did not significantly alter *Pde10a* mRNA levels in striatal subregions or the neighboring lateral septum**. **(A)** *Pde10a* mRNA expression was not significantly modified in rats acutely (8–10 h) withdrawn from intermittent alcohol vapor (black bars), as compared to alcohol-naïve controls (white bars), in the nucleus accumbens, dorsal striatum, or lateral septum. **(B)** *Pde10a* mRNA levels remained unaltered in the nucleus accumbens and lateral septum during protracted (6 weeks) alcohol withdrawal, although levels tended to be reduced in the dorsal striatum of alcohol-withdrawn rats. Data are presented as least squares means ± standard error of *z*-score transformed *Pde10a* covaried for *Cyp* expression levels. *n* = 8–12 per group.

Despite, or perhaps due to, the striatum displaying the highest neuronal expression of PDE10A (Seeger et al., 2003), no significant changes in *Pde10a* mRNA levels were observed in the dorsal striatum (DS) or nucleus accumbens (NAc) during acute withdrawal (**Figure 3A**, *F*’s < 0.37, *p*’s > 0.55). During protracted alcohol withdrawal (**Figure 3B**), *Pde10a* mRNA expression tended to be reduced in the DS (*F*_1,20_ = 4.09, *p* = 0.057) but not NAc (*F*_1,19_= 0.29, *p* = 0.60). The neighboring lateral septum (LS), a brain region previously shown to be activated during alcohol withdrawal (Knapp et al., 1998; Kozell et al., 2005), displayed no significant alterations in *Pde10a* mRNA expression during acute (**Figure 3A**, *F*_1,__2__1_ = 1.58, *p* = 0.22) or protracted (**Figure 3B**, *F*_1,19_ = 0.38, *p* = 0.55) alcohol withdrawal.

### RELATIVE *Pde10a* mRNA EXPRESSION AMONG TESTED BRAIN REGIONS

**FIGURE 4:**
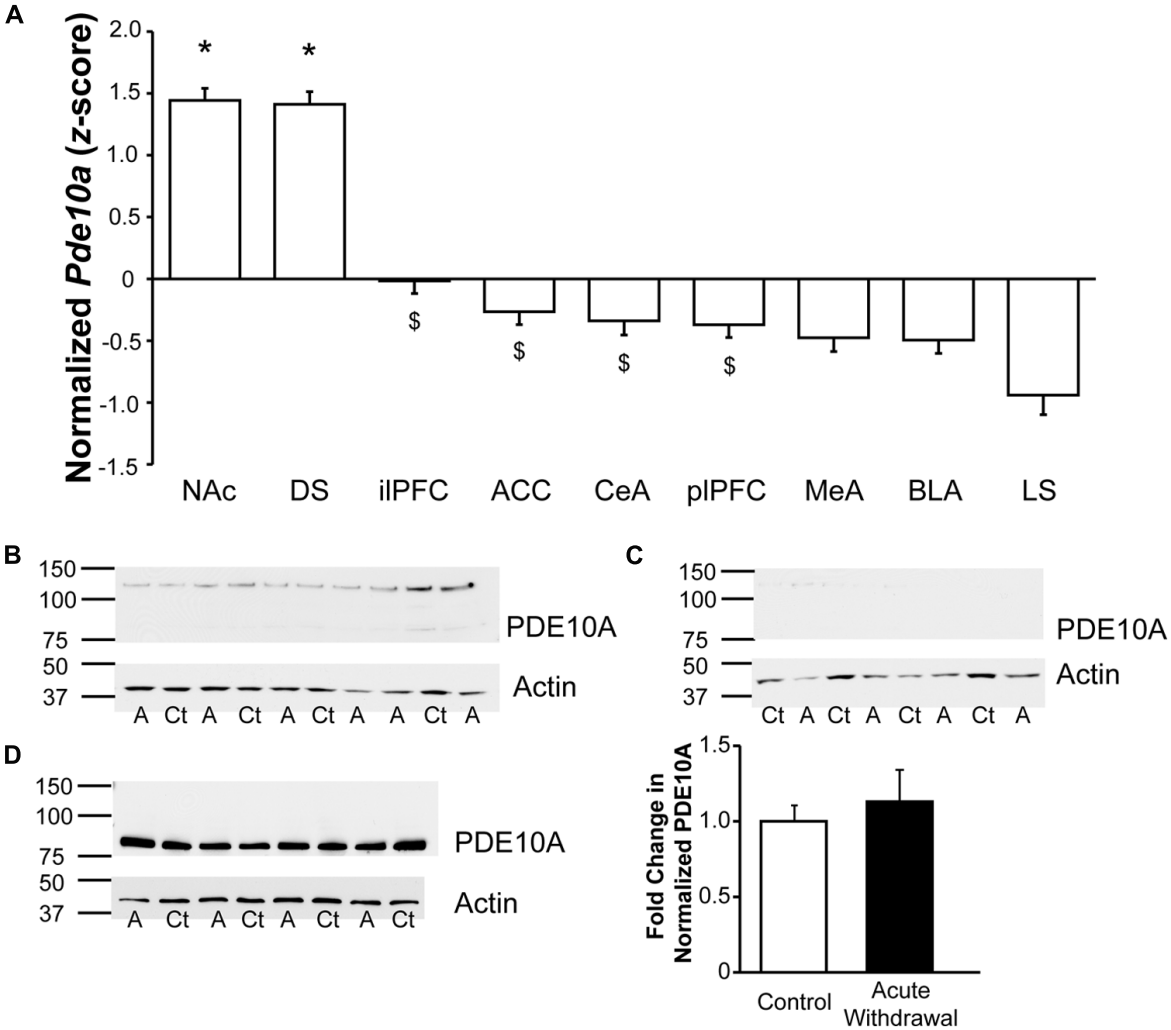
**Significant enrichment in striatal *Pde10a* expression relative to other stress- and reward-responsive brain regions**. **(A)** Quantitative analysis of regional *Pde10a* mRNA expression in alcohol-naïve controls demonstrated very significantly lower *Pde10a* expression in medial prefrontal and amygdala subdivisions, as compared to the striatal regions (DS, NAc). Of the extrastriate regions assessed, infralimbic prefrontal cortex demonstrated the highest and lateral septum the lowest levels of *Pde10a* mRNA in alcohol-naïve control rats. NAc, nucleus accumbens; DS, dorsal striatum; ilPFC, infralimbic prefrontal cortex; ACC, anterior cingulate cortex; CeA, central amygdala; plPFC, prelimbic prefrontal cortex; MeA, medial amygdala; BLA, basolateral amygdala; LS, lateral septum. Data are presented as least squares means ± standard error of *z*-score transformed *Pde10a* covaried for *Cyp* expression levels. *p < 0.05 vs. extrastriatal regions, ^$^*p* < 0.05 vs. lateral septum; *n* = 21–24 per region. **(B–D)** PDE10A protein levels, as compared to β-actin expression, were assessed using Western blotting techniques. Numbers to the left of membrane images depict the position of molecular weight markers (in kDa), while letters beneath the lanes indicate treatment groups (Ct, control; A, alcohol-withdrawn). **(B)** Lysate of protracted withdrawal BLA samples displayed very low levels of the 88 kDa PDE10A protein signal such that reliable quantification was not possible. **(C)** A postsynaptic density-enriched fraction from protracted withdrawal BLA also showed minimal levels of PDE10A protein immunostaining. **(D)** High levels of PDE10A protein immunostaining were observed in the dorsal striatum postsynaptic density-enriched fraction obtained during acute withdrawal, without significant differences between alcohol-naïve (Control) and acutely Alcohol Withdrawn rats. Histogram depicts the quantification of PDE10A protein levels normalized to β-actin, expressed as the fold change relative to mean normalized PDE10A levels in the control group. *n* = 4 per group.

Comparison of regional *Pde10a* expression (**Figure 4A**) via qPCR in samples obtained from controls demonstrated significant regional differences (*F*_8,192_** = 50.42, *p* < 0.001), confirming the marked striatal *Pde10a* mRNA enrichment previously reported (Fujishige et al., 1999b; Kelly et al., 2014) relative to all other regions tested. Of the extra-striatal regions, the comparatively lower expression was greatest in the infralimbic prefrontal cortex and lowest in the LS (**Figure 4A**).

Consistent with the much lower levels of *Pde10a* mRNA expression in the amygdala, Western blot analysis was not sufficiently sensitive to yield adequate PDE10A protein signal at the 88 kDa band for reliable quantitation in either whole tissue lysates (**Figure 4B**) or in postsynaptic membrane-enriched samples (**Figure 4C**) from the BLA, unlike dorsal striatal membrane-enriched samples (**Figure 4D**), where PDE10A expression was not altered during acute withdrawal, consistent with the mRNA data. It should be noted that PDE10A protein expression was visible in BLA immunoblots, albeit at very low levels that were not suitable for quantitation. The quantity of protein loaded in the present study was consistent with other fractionation studies (Kotera et al., 2004; Fumagalli et al., 2008; Pacchioni et al., 2009). Still, a greater protein load might have led to a different result and could conceivably be achieved by sample pooling or other methods of protein enrichment. Limits of extracted protein concentration and well volumes prevented greater loads in the present study.

## DISCUSSION

Acute and protracted withdrawal from intermittent alcohol vapor exposure generated neuroanatomically distinct profiles of altered *Pde10a* mRNA expression in the rat. Acute withdrawal produced widespread elevations in *Pde10a* levels in stress- and reward-responsive nuclei of the limbic system. This effect was particularly striking in the interconnected subdivisions of the amygdala and mPFC (Vertes, 2004; Hoover and Vertes, 2007), as *Pde10a* expression was increased in the BLA, MeA, and CeA subdivisions of the amygdala, as well as in the ilPFC and ACC subdivisions of the mPFC. *Pde10a* levels were unaltered in regions with the highest baseline *Pde10a* expression, namely the DS and NAc, as well as in the neighboring LS, though it should be noted that high baseline striatal *Pde10a* expression levels may preclude observation of small changes that would easily attain significance in brain regions with lower baseline expression. Regardless, these data suggest that acute alcohol withdrawal may recruit PDE10A activity in regions with otherwise low basal expression, perhaps as a compensatory mechanism to reduce heightened neural activity observed during acute withdrawal in the amygdala and mPFC (Knapp et al., 1998; Roberto et al., 2004; Lack et al., 2007; Zhu et al., 2007; George et al., 2012; Kroener et al., 2012).

Following an extended, 6-weeks period of abstinence, *Pde10a* mRNA expression levels remained heightened only in the BLA. Interestingly, elevated BLA *Pde10a* mRNA also has been seen in rats with a history of stress and alcohol self-administration, as compared to stress-naïve but similarly alcohol-experienced controls (Logrip and Zorrilla, 2012). The collective data suggest that stressful experiences, including repeated cycles of withdrawal from intoxicating alcohol exposure, repeated exposure to mild footshock (Logrip and Zorrilla, 2012), or their combination, may generate long-lasting adaptations in *Pde10a* mRNA expression levels. A putative behavioral function of elevated BLA PDE10A protein levels might be to modulate stress-related behaviors because both footshock (Kinn Rod et al., 2012) and withdrawal from alcohol (Zhao et al., 2007; Sommer et al., 2008) can result in heightened anxiety-like behavior for days or weeks after the stressful experience. A previous study attributed anxiogenic-like behavior during acute withdrawal from alcohol liquid diet to reduced cAMP signaling in the CeA, but not BLA (Pandey et al., 2003), an effect that could result from elevated PDE10A levels. Accordingly, the present study observed a trend (*p* = 0.053) for elevated CeA *Pde10a* mRNA levels during acute withdrawal. In addition, however, the present study also observed lasting increases in BLA *Pde10a* expression. This regional discrepancy may result from differences in alcohol withdrawal time point (24 h vs. 8–10 h or 6 weeks withdrawal) or in the alcohol exposure paradigm (2 weeks continuous liquid diet vs. 5 weeks intermittent alcohol vapor). Alternatively, because PDE10A can decrease the activity of both cAMP- and cGMP-dependent signaling cascades, BLA PDE10A might modulate anxiety-like behavior by reducing cGMP activity, alone or in combination with cAMP signaling.

Systemic inhibition of PDE10A by pharmacological (Siuciak et al., 2006a; Schmidt et al., 2008; Grauer et al., 2009) or genetic (Siuciak et al., 2006b) methods increases the latency to exit a shock-paired chamber and attenuates amphetamine-induced deficits in auditory gating, further supporting a possible role for PDE10A in modulating stress-responsive behaviors. The BLA plays a prominent role in emotional memory processing (Laviolette et al., 2005; Laviolette and Grace, 2006; Stuber et al., 2011) for both appetitive and aversive stimuli (Dwyer, 2011), and in light of the present data, the BLA is hypothesized to be a site of action via which PDE10A regulates stress-related behavior, perhaps by enacting lasting changes in BLA neuronal activity. Altered BLA activity could produce many behavioral effects based on numerous efferent projections. In addition to the CeA, the output region of the amygdala (Pitkanen et al., 1995; Savander et al., 1995), significant direct BLA outputs have been demonstrated to the NAc (Russchen and Price, 1984; McDonald, 1991, 1992), mPFC (Krettek and Price, 1977; McDonald, 1987, 1991, 1992), hippocampus (Pitkanen et al., 1995; Savander et al., 1995), and bed nucleus of the stria terminalis (Weller and Smith, 1982), all regions which may modulate reward-, withdrawal-, and stress-related behaviors. Thus, understanding how PDE10A may participate in alcohol withdrawal-associated BLA plasticity is of great interest for long-term adaptations in circuit-wide reward and stress responses.

It is of particular interest to note similarities in the profiles of regional alteration in *Pde10a* mRNA expression during acute alcohol withdrawal and those we previously reported in a behavioral model of stress history elevation of relapse-like alcohol self-administration (Logrip and Zorrilla, 2012), as elevated alcohol self-administration is observed during acute withdrawal from intermittent alcohol vapor exposure (Rimondini et al., 2003; O’Dell et al., 2004; Finn et al., 2007). Analysis of *Pde10a* expression at the conclusion of behavioral testing demonstrated several interesting relationships to parameters of alcohol self-administration: BLA *Pde10a* levels positively correlated with alcohol preference and CeA *Pde10a* positively correlated with alcohol intake in high and low drinkers, respectively, during acquisition of operant responding. Similarly, ilPFC *Pde10a* levels directly correlated with the level of relapse-like alcohol self-administration in rats with very high levels of intake, while plPFC *Pde10a* levels directly correlated with relapse-like alcohol intake in rats with low baseline levels of alcohol self-administration, the group showing the greatest stress history-induced increase in relapse-like self-administration. Given the elevated alcohol self-administration and preference observed in alcohol-dependent rats (Rimondini et al., 2003; O’Dell et al., 2004) and the increased *Pde10a* observed during acute withdrawal in the BLA, CeA, and ilPFC, these data suggest a possible role for withdrawal-induced elevations in amygdala and mPFC PDE10A in generating increased alcohol intake in alcohol-dependent rats.

### PDE10A AS A MODULATOR OF NEURONAL RESPONSIVENESS

The mechanism by which withdrawal-induced PDE10A may alter neuronal activity, thereby contributing to long-term behavioral adaptations, remains unclear. As a negative regulator of both cAMP and cGMP signaling (Fujishige et al., 1999a), PDE10A is poised to regulate several intracellular signaling cascades, thereby playing a key role in neuronal responses to stimuli. Indeed, pharmacological inhibition of PDE10A increases the striatal response to stimulation of cortical inputs (Threlfell et al., 2009), implicating PDE10A as a regulator of neuronal activity. A possible long-term mechanism for PDE10A modulation of neuronal activity involves PKA- or PKG-dependent regulation of AMPA receptor membrane insertion via phosphorylation of the GluA1 subunit (Man et al., 2007; Serulle et al., 2008). PDE10A inhibitor treatment increases the phosphorylation of GluA1 at serine residue 845 (Nishi et al., 2008; Grauer et al., 2009), including at the cell surface (Grauer et al., 2009), indicating that elevated PDE10A expression may reduce GluA1 phosphorylation and thereby the synaptic expression of GluA1-containing AMPA receptors. Genetic reduction of GluA1 phosphorylation decreases anxiety-like behavior in rats (Kiselycznyk et al., 2013), whereas chronic alcohol exposure/withdrawal increases BLA GluA1 phosphorylation (Christian et al., 2012). Insofar as we observed elevated BLA *Pde10a* levels during protracted abstinence time points associated with heightened anxiety-like behavior (Valdez et al., 2002; Zhao et al., 2007), it might be argued that the elevated PDE10A expression represents a compensatory response. Alternatively, PDE10A’s net action on neuronal responsivity in the BLA may not occur primarily via AMPA receptor subunit GluA1 phosphorylation and membrane insertion. Inhibition of PDE10A activity alters the phosphorylation state of other proteins besides GluA1 (Nishi et al., 2008), as well as the expression of several neurotransmission-related genes (Kleiman et al., 2011). Thus, PDE10A regulation of neuronal activity may also occur via an intracellular pathway, independent of the synaptic expression of AMPA receptors. Determining the molecular consequences of elevated BLA PDE10A presents an intriguing future endeavor.

A key unresolved question is whether the increase in BLA PDE10A expression occurs in the predominant population of glutamatergic pyramidal cells, or, instead, in the minority GABAergic interneuron population (McDonald, 1982, 1985). For example, if PDE10A dampens neuronal activity in the BLA as it does in the PDE10A-rich striatum (Threlfell et al., 2009; Mango et al., 2014), then increased PDE10A expression in BLA GABAergic interneurons would reduce their activity, leading to disinhibition of glutamatergic pyramidal cells and greater excitability of BLA efferents. In contrast, increased PDE10A expression in BLA pyramidal neurons would reduce their excitability and excitatory glutamatergic outflow. The neuroanatomical localization of increased BLA PDE10A during withdrawal may thus yield insights as to whether PDE10A contributes to or serves as a compensatory response to the heightened glutamatergic synaptic transmission that has been reported in the BLA following chronic intermittent alcohol exposure (Floyd et al., 2003; Lack et al., 2007, 2009; Christian et al., 2012, 2013).

## CONCLUSION

Rats undergoing acute, but not protracted, withdrawal from alcohol showed widespread elevations in *Pde10a* mRNA expression in interconnected mPFC and amygdala subdivisions that could regulate the elevated self-administration observed in alcohol-dependent rats. The persistence of elevated *Pde10a* in the BLA 6 weeks after the final alcohol exposure implicates lasting changes in BLA neuronal activity as a possible factor in the persistence of relapse propensity following cessation of alcohol use. Future investigation into the mechanisms by which PDE10A modulates BLA activity and the role of amygdala PDE10A in regulating anxiety- and alcohol-related behaviors is of interest for developing therapies to treat both alcohol use and stress-related disorders.

## Conflict of Interest Statement

The authors declare that the research was conducted in the absence of any commercial or financial relationships that could be construed as a potential conflict of interest.
